# Distinct domains of *Escherichia coli* IgaA connect envelope stress sensing and down-regulation of the Rcs phosphorelay across subcellular compartments

**DOI:** 10.1371/journal.pgen.1007398

**Published:** 2018-05-31

**Authors:** Nahla A. Hussein, Seung-Hyun Cho, Géraldine Laloux, Rania Siam, Jean-François Collet

**Affiliations:** 1 WELBIO, Brussels, Belgium; 2 de Duve Institute, Université catholique de Louvain, Brussels, Belgium; 3 Biology Department, Biotechnology Graduate Program and YJ-Science and Technology Research Center, American University in Cairo, Cairo, Egypt; University of Geneva Medical School, SWITZERLAND

## Abstract

In enterobacteria, the Rcs system (Regulator of capsule synthesis) monitors envelope integrity and induces a stress response when damages occur in the outer membrane or in the peptidoglycan layer. Built around a two-component system, Rcs controls gene expression *via* a cascade of phosphoryl transfer reactions. Being particularly complex, Rcs also involves the outer membrane lipoprotein RcsF and the inner membrane essential protein IgaA (Intracellular growth attenuator). RcsF and IgaA, which are located upstream of the phosphorelay, are required for normal Rcs functioning. Here, we establish the stress-dependent formation of a complex between RcsF and the periplasmic domain of IgaA as the molecular signal triggering Rcs. Moreover, molecular dissection of IgaA reveals that its negative regulatory role on Rcs is mostly carried by its first N-terminal cytoplasmic domain. Altogether, our results support a model in which IgaA regulates Rcs activation by playing a direct role in the transfer of signals from the cell envelope to the cytoplasm. This remarkable feature further distinguishes Rcs from other envelope stress response systems.

## Author summary

A thorough understanding of the mechanisms that allow bacteria to thrive in various environments is crucial to the development of new antibiotics, an urgent endeavor to combat antimicrobial resistance. A landmark feature of Gram-negative bacteria is the presence of a multi-layered envelope. Because this structure is essential, its integrity is constantly monitored to detect and respond to potential breaches in a fast and adequate manner. Here, we describe how IgaA, an essential protein present in the cytoplasmic membrane of enterobacteria, participates in the transfer of stress signals from the envelope to the cytoplasm. We provide evidence that IgaA works in concert with RcsF, a lipoprotein that is posted as a sentinel in the outermost envelope layer, to detect envelope stress: under stress conditions, RcsF forms a complex with the C-terminal, periplasmic domain of IgaA. As a result, cells turn on the Rcs response. We also discovered that the N-terminal, cytoplasmic domain of IgaA plays an important role in inhibiting Rcs in the absence of stress. Together, these findings reveal that distinct IgaA domains coordinate stress sensing and Rcs activation across the cytoplasmic membrane. They enhance our understanding of Rcs regulation and open new avenues for the development of new antibacterials.

## Introduction

Gram-negative bacteria are surrounded by the cell envelope, a multi-layered structure composed of an outer membrane (OM) and an inner membrane (IM). These two membranes delimit the periplasm, a viscous and oxidizing compartment enclosing the cell wall, a thin peptidoglycan (PG) layer. The cell envelope is required for growth and survival, maintaining cell shape and providing osmotic protection to cells [1]. Being at the interface with the environment, the envelope is also a permeability barrier protecting bacteria from environmental stress and antibacterial compounds [2]. Proteins playing a role in the assembly and maintenance of the cell envelope are therefore attractive targets for antibiotic development.

**Fig 1:**
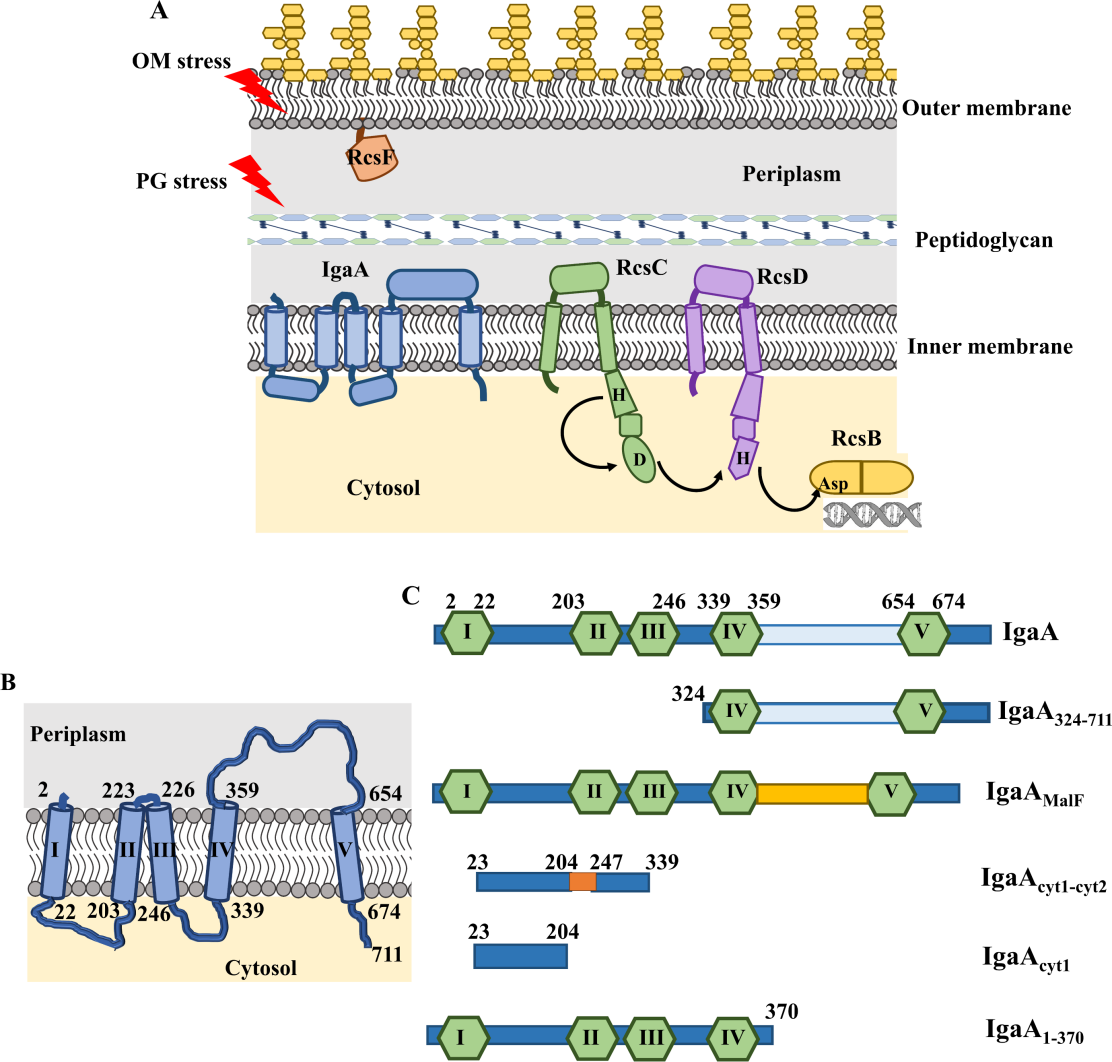
Multiple components of the Rcs phosphorelay system. (A) Diagrammatic representation of the Rcs system. The Rcs system is formed of the histidine kinase RcsC, the phosphotransfer protein RcsD and the cytosolic response regulator RcsB. Two auxiliary proteins are also associated with the Rcs system: the outer membrane lipoprotein sensor RcsF and the negative regulator inner membrane protein IgaA [5]. For simplicity, RcsF is represented as a free OM lipoprotein facing the periplasm. We do not show its β-barrel interacting partners that allow portions of RcsF to reach the cell surface. Black arrows represent the direction of phosphotransfer from RcsC to RcsD and finally to RcsB. RcsB binds as homodimers or heterodimers (with RcsA, not represented in the figure) to regulate numerous genes including those involved in colanic acid capsule synthesis, motility, sRNA, etc [5]. (B) Predicted topology of IgaA, the negative regulator of the Rcs system. The topology of *E*. *coli* YrfF (IgaA, uniprot ID P45800) was predicted according to the TOPCONS server [40]. IgaA structure shows the presence of five transmembrane domains, two cytosolic domains at the N-terminus and one periplasmic domain close to the C-terminus. Roman numbers indicate the transmembrane domains. Arabic numbers denote amino acid positions at the beginning and end of each TM domain according to the consensus topology predicted by TOPCONS. (C) Schematic representations of the IgaA variants generated during this study. All constructs were generated with a C-terminal tag of either penta-histidine (His) or triple flag (fl). TM segments are represented by green hexagons while the N-terminal cytosolic parts are schematized as dark blue rectangles. The C-terminal periplasmic domain of IgaA is represented by a light blue rectangle while the MalF periplasmic domain is represented as an orange rectangle.

Given the functional and structural importance of the envelope, it is a matter of life and death for bacteria to detect breach in envelope integrity and to respond in a fast and adequate manner. Bacteria have therefore evolved sophisticated systems that allow them to monitor envelope integrity and to elicit cellular responses when perturbations occur [3]. In *Escherichia coli* and enterobacteria, the Rcs system (**Fig 1A**) detects a variety of envelope perturbations, the most prominent being OM and PG damage [4,5]. In response, Rcs modulates the expression of dozens of genes, including those involved in the biosynthesis of colanic acid, an exopolysaccharide that accumulates on the cell surface to form a protective capsule [5,6]. In addition to its role in capsule formation, Rcs is also required for normal biofilm development and regulates virulence-associated structures [5,7].

Like several envelope stress sensing systems, Rcs is built around a two-component system. In classical two-component systems, an IM-localized histidine kinase autophosphorylates on a histidine residue in response to a specific signal. The phosphoryl group is then transferred to an aspartate present in a cytoplasmic response regulator, which then binds to target promoters on the chromosome to control gene transcription [5]. In Rcs, the phosphorylation cascade is however more complex. Indeed, following autophosphorylation of the sensor histidine kinase RcsC, the phosphoryl group is first transferred to an aspartate residue present in a receiver domain on the same protein. It is then being handed over to a histidine residue present in the IM protein RcsD, before being finally transferred to an aspartate present in the receiver domain of the response regulator RcsB (**Fig 1A**). Thus, in the Rcs system, signal transduction involves a multi-step phosphorelay [8–10].

A second unusual feature of Rcs is that proteins that do not directly participate in the phosphorylation cascade modulate the activity of the system. It is the case of RcsF, an OM lipoprotein that is at least partially exposed on the cell surface [11–14]. Surface exposure of RcsF is mediated by the β-barrel assembly machinery via the assembly of complexes between RcsF and abundant β-barrels [11,12]. Interestingly, RcsF is required for sensing most Rcs-inducing cues, including OM alterations by cationic antimicrobial peptides [15] or weakening of the PG sacculus by mecillinam, a β-lactam antibiotic inhibiting the essential transpeptidase PBP2 [4]. A second auxiliary protein that is important for Rcs function is YrfF (**Fig 1B**), a poorly abundant IM protein that down-regulates Rcs *via* a still unknown mechanism. YrfF has been mainly studied in *Salmonella*, where it was found to be implicated in pathogenicity and antimicrobial resistance [16–19]. Because *yrfF* inhibits growth of *Salmonella* inside fibroblasts, it was renamed to IgaA (for Intracellular growth attenuator) [18]. We will adopt this nomenclature here for the *E*. *coli* gene. Interestingly, *igaA* is the only gene encoding an Rcs component that is essential [20], indicating that excessive Rcs activation is toxic for cells. Notably, *igaA* null alleles are only viable when combined with deletions of *rcsB*, *rcsC* and *rcsD*, but not *rcsF*, which implies that IgaA lies upstream of the components of the phosphorelay and downstream of RcsF in the signaling cascade [11,20,21].

We previously reported that, when the periplasmic domain of IgaA is expressed as a soluble protein, it forms complexes with RcsF which can be pulled-down after cross-linking [11]. The interaction between these two proteins was also confirmed *in vitro* [11] and by using bimolecular fluorescence complementation [22]. These and other results led us to propose a model in which OM or PG-related stress prevents newly synthesized RcsF from interacting with the β-barrel assembly machinery, which results into new RcsF molecules being exposed to the periplasm, where they bind to IgaA. Following interaction with RcsF, IgaA would then relieve the inhibition that it exerts on the phosphorylation cascade, turning on Rcs [11]. However, direct evidence for the stress-dependent formation of the RcsF-IgaA complex is still missing. Furthermore, nothing is known on how IgaA interacts with the downstream Rcs components and regulates the phosphorelay.

Here, we clearly established the functional relevance of the RcsF-IgaA interaction by obtaining direct evidence for the stress-induced formation of the RcsF-IgaA complex. In addition, by testing the ability of a series of IgaA constructs corresponding to different domains of the protein to complement an *igaA* depletion strain, we functionally dissected IgaA to gain insights into its mechanism of action. We found that while the C-terminal, periplasmic domain of IgaA serves as the primary receiver of the signal transmitted by RcsF, it is not required for Rcs inhibition. By contrast, substantial Rcs inhibition was observed when the first cytoplasmic domain of IgaA was expressed as a soluble protein, revealing the important role of this domain in Rcs regulation. Full Rcs repression required, however, co-expression of the N-terminal and C-terminal portions of IgaA. Altogether, our results establish IgaA as a multimodal platform capable of integrating signals on both sides of the IM.

## Results

### IgaA forms a stress-dependent complex with the OM lipoprotein RcsF

The functional importance of the RcsF-IgaA interaction and its role in turning on Rcs under stress remained unclear. To close this gap, we engineered a molecular system to monitor the formation of the RcsF-IgaA complex *in vivo* and determine its levels under different conditions. Because IgaA is a relatively low abundant protein, being present at ~200 copies per cell [23], we expressed it from a low-copy plasmid to increase its expression levels and facilitate detection. A triple flag tag (denoted as fl) was fused to the C-terminus of the protein (IgaA-fl) for purification and detection purposes. The fact that Δ*igaA* cells expressing IgaA-fl are viable (**S1 Fig**) indicated that the fusion protein, which correctly localizes to the IM (**S2A Fig**), is functional.

**Fig 2:**
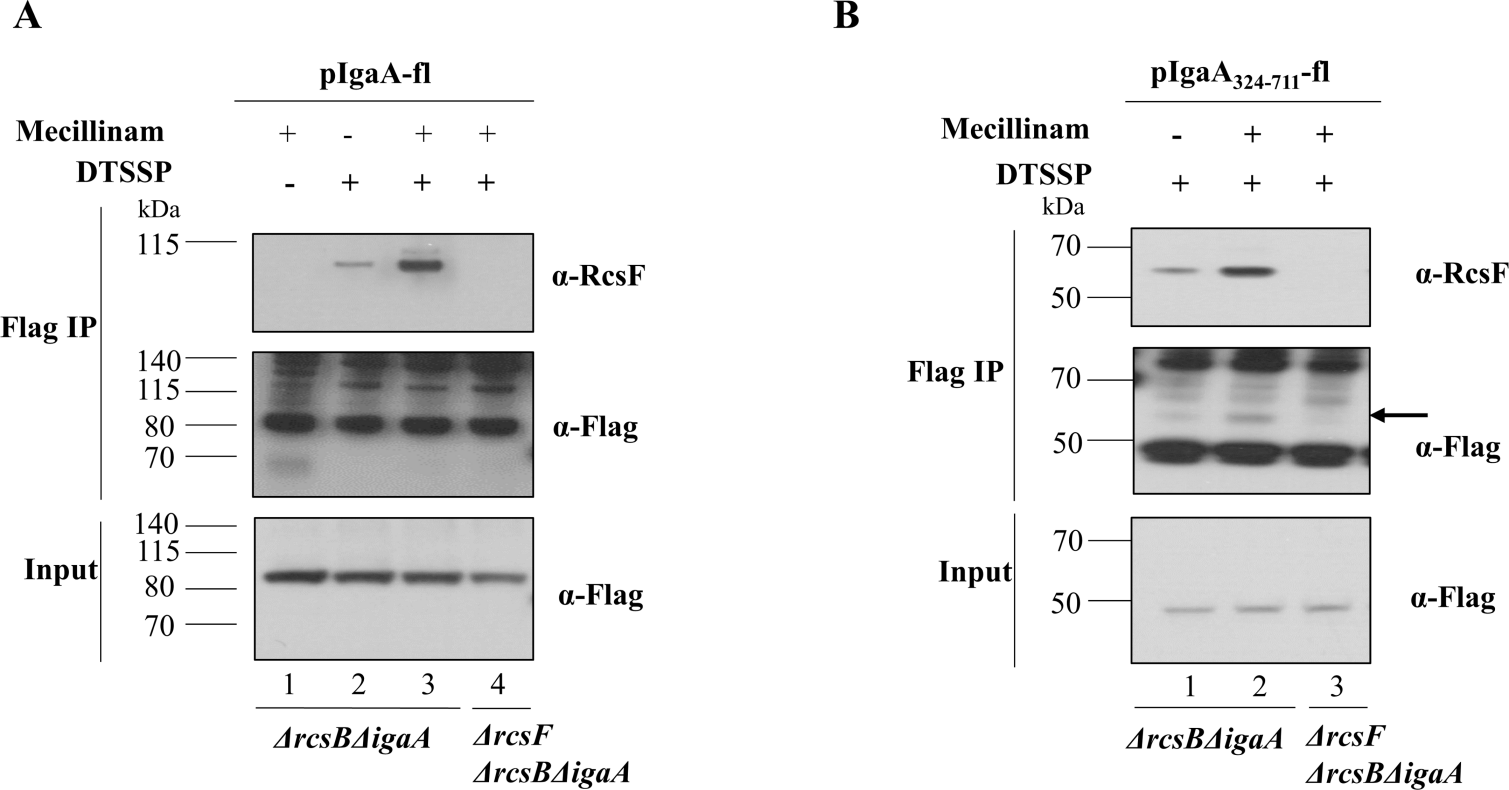
RcsF interacts with the periplasmic domain of IgaA. (A) Complex formation between IgaA and RcsF occurs even in the absence of stress and increases in response to mecillinam treatment. Expression of pIgaA-fl (from pSC238) was induced with 100 μM IPTG in *ΔrcsBΔigaA* or *ΔrcsFΔrcsBΔigaA* cells. Cells were treated with 0.3 μg/ml mecillinam for one hour, cross-linked with DTSSP and immunoprecipitated using flag-coated magnetic beads. The ~ 100 kDa RcsF-IgaA complex was only detected with the anti-RcsF antibody (lane 2) and increased upon mecillinam treatment (lane 3). Complex formation was not observed in the negative controls (no DTSSP, lane 1; *ΔrcsFΔrcsBΔigaA* cells, lane 4). The bands detected at ~140 kDa and ~115 kDa by the anti-flag antibody correspond to mouse IgG (eluted during the immunoprecipitation) and unknown cross-reactive proteins, respectively. (B) RcsF specifically interacts with the C-terminal periplasmic domain of IgaA. The same procedure was followed as in (A) but using pIgaA_324-711_-fl (from pNH441). The ∼60 kDa RcsF-IgaA_324-711_ complex could be detected with anti-flag (indicated by the black arrow) and anti-RcsF antibodies and increased in the presence of mecillinam. The complex could only form in *ΔrcsBΔigaA* (lanes 1 and 2) but not in the *ΔrcsBΔrcsFΔigaA* cells (lane 3). Data shown in (A) and (B) are representative of three biological replicates.

We first decided to determine whether RcsF interacts with IgaA in non-stressed cells, as suggested by the fact that basal Rcs activity was measured in cells grown under normal conditions (**S3 Fig**). To that end, IgaA-fl was expressed in Δ*rcsB*Δ*igaA* cells and the water soluble cross linker 3,3'-dithiobis(sulfosuccinimidylpropionate) (DTSSP) was added. We used cells deleted for *rcsB* to prevent Rcs induction, which could otherwise modify cellular permeability to the crosslinker (for instance via capsule production) and influence results. After immunoprecipitation with beads conjugated with the anti-flag antibody, a band of ∼100 kDa, the size expected for the IgaA-fl (82 kDa)-RcsF (14 kDa) complex, was detected by immunoblotting with an anti-RcsF antibody in the DTSSP-treated sample (lane 2, **Fig 2A**). This band was not observed in cells lacking *rcsF* (lane 4, **Fig 2A**), indicating that it most likely corresponded to the IgaA-RcsF complex.

To provide further experimental support for this identification, the *in vivo* cross-linking experiment was repeated using cells expressing a truncated variant of IgaA (from S324 to E711, here referred as IgaA_324-711_-fl) corresponding to the periplasmic domain of the protein anchored to the IM (**S2B Fig**) *via* transmembrane (TM) segments IV and V (**Fig 1C**). The expression of IgaA_324-711_-fl was induced by addition of IPTG in *ΔrcsBΔigaA* and *ΔrcsBΔigaAΔrcsF* cells. After pull-down, a band of ∼60 kDa corresponding to the size expected for a complex between IgaA_324-711_-fl (45 kDa) and RcsF (14 kDa) was detected both by the anti-RcsF and the anti-Flag antibodies (lane 1, **Fig 2B**). This band was observed in Δ*rcsB*Δ*igaA* cells treated with DTSSP, but not in Δ*rcsB*Δ*igaA*Δ*rcsF* (lane 3, **Fig 2B**). Thus, our data clearly established that RcsF and IgaA interact *in vivo*, even in the absence of stress. They also indicated that the C-terminal periplasmic domain of IgaA is sufficient to mediate the interaction.

We next asked whether exposure to stress would increase the levels of the RcsF-IgaA complex, as expected if this interaction serves as the molecular signal triggering Rcs. To that purpose, we monitored complex formation in cells treated with mecillinam, a β-lactam antibiotic that inhibits the essential transpeptidase PBP2 [24,25] and activates the Rcs system in an RcsF-dependent manner [4]. Remarkably, the 100 kDa-band corresponding to RcsF-IgaA substantially increased following mecillinam treatment (lane 3, **Fig 2A**). A similar increase was observed for the ~60 kDa-band in cells expressing the truncated protein IgaA_324-711_-fl (lane 2, **Fig 2B**). Thus, these results demonstrate for the first time the increased formation of the RcsF-IgaA complex in response to Rcs-inducing stress, providing crucial experimental support to the model that the RcsF-IgaA interaction controls Rcs activation.

### Construction of an *igaA* depletion strain for the molecular dissection of IgaA

According to topology models, IgaA is inserted in the IM *via* 5 TM segments (**Fig 1B**). These segments determine two N-terminal cytoplasmic domains of 21 and 11 kDa, respectively, separated by a short periplasmic connector consisting of three amino acids residues, and a C-terminal periplasmic domain of 295 residues. Thus, the N-terminal part of IgaA comprised between TMI and TMIV appears to be mostly exposed to the cytoplasm, while the C-terminal portion is mainly periplasmic (**Fig 1B**).

Nothing is known on how IgaA modulates Rcs activity. Because both RcsC and RcsD possess a large periplasmic domain (**Fig 1A**), it is possible that IgaA inhibits Rcs by interacting with one or both proteins in the periplasm. In this case, conformational changes in the periplasmic domain of IgaA upon formation of a complex with RcsF would, in turn, alleviate the inhibition on RcsC and/or RcsD, turning on Rcs. Alternatively, RcsF binding in the periplasm may trigger conformational rearrangements in the cytoplasmic part of IgaA that would then be sensed by RcsC and/or RcsD in this compartment. In this second scenario, IgaA inhibits Rcs via its cytoplasmic domain. These two models are not mutually exclusive, and it is possible that both the periplasmic and cytoplasmic portions of IgaA contribute to Rcs regulation.

**Fig 3:**
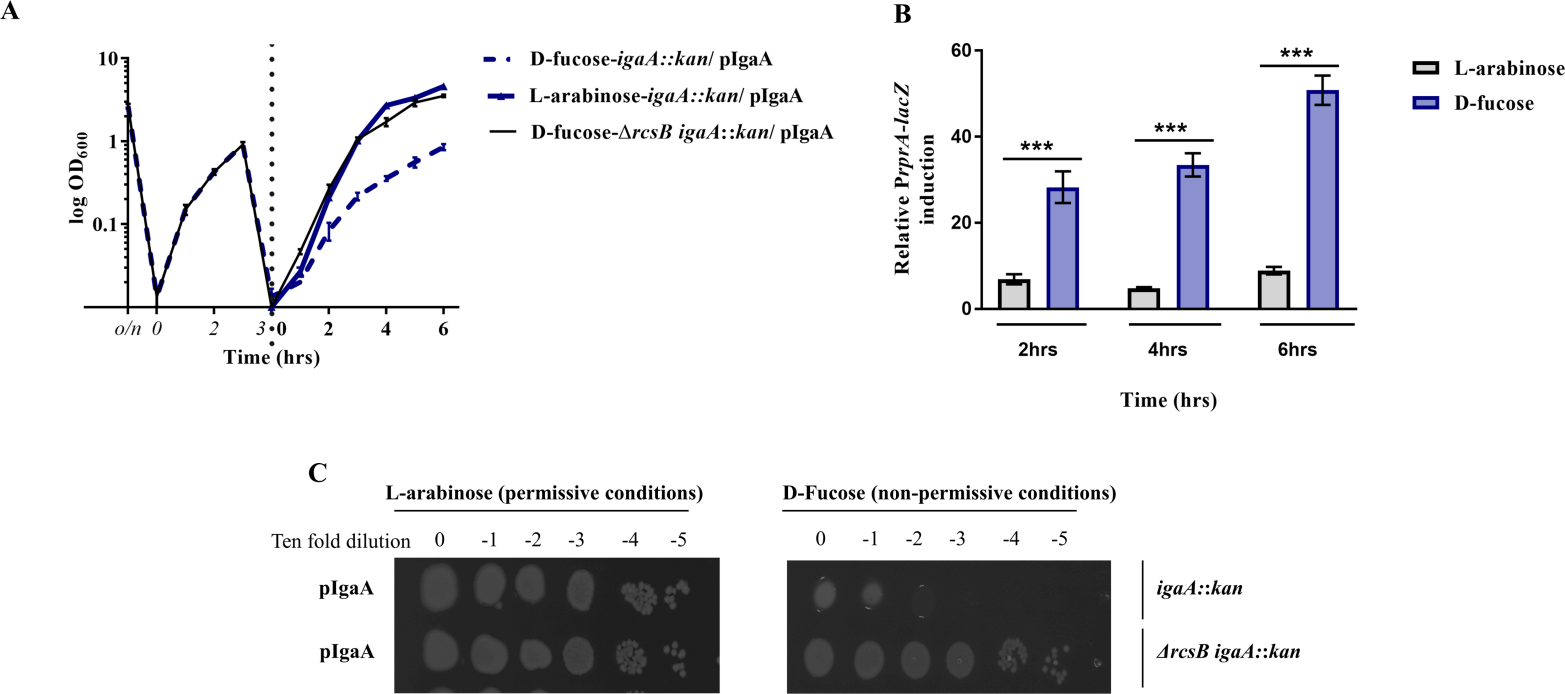
Growth impairment of the *igaA* depletion strain correlates with activation of the Rcs system. (A) *E*. *coli* growth is impaired after a two-phases depletion of *igaA*. Strains expressing IgaA-His (from pNH586) in either *igaA*::*kan* or Δ*rscBigaA*::*kan* cells were subjected to an initial depletion by culturing in the presence of 0.2% D-fucose for ~ 8 generations (italicized time points, left of the dotted line). During this phase, growth of the *igaA*::*kan* strain was similar to that of the Δ*rcsBigaA*::*kan* strain, indicating that the Rcs system was efficiently repressed, despite undetectable IgaA levels (**S5 Fig**). When the cells were sub-cultured in fresh media containing either 0.2% L-arabinose (permissive) or 0.2% D-fucose (non-permissive), only the *igaA*::*kan* strain cultured under the non-permissive conditions showed a pronounced growth defect, confirming that *igaA* was efficiently depleted under these conditions. Data shown represent the mean of three biological replicates, error bars represent the standard error of the mean (SEM). (B) The Rcs system is activated upon *igaA* depletion. After *igaA* initial depletion, cells were diluted in fresh LB containing either 0.2% L-arabinose or 0.2% D-fucose. Rcs activation was periodically monitored by assessing P*rprA*-*lacZ* activity [36]. Data shown here correspond to the Rcs system activation measured in *igaA*::*kan* cells harboring pNH586 and cultured in the presence of either L-arabinose or D-fucose. Data were normalized by Rcs activity measured in Δ*rscBigaA*::*kan* cells treated similarly. Indicated time points correspond to time after *igaA* initial depletion. Values represent the mean of three biological replicates. Error bars represent standard error of the mean (SEM). For all panels, ** P≤ 0.01 and *** P≤ 0.001. (C) After an initial *igaA* depletion, strains expressing IgaA-His (from pNH586) in either *igaA*::*kan* or Δ*rscBigaA*::*kan* backgrounds were serially diluted and spotted on LB-agar plates supplemented with either 0.2% L-arabinose (permissive conditions) or 0.2% D-fucose (non-permissive conditions). Plates shown are representative of 3 independent experiments.

To obtain insights into the mechanism used by IgaA to regulate Rcs, we decided to molecularly dissect this protein to investigate the different roles of its periplasmic and cytoplasmic portions. However, before proceeding with further experiments, we first generated an *igaA* depletion strain because of the essentiality of *igaA* [20]. To that purpose, an L-arabinose-inducible copy of *igaA* on a medium copy-number vector was transformed into a wild-type strain. This strain also carried an *rprA*::*lacZ* fusion on the chromosome to monitor Rcs activity [25]. Then, the chromosomal copy of *igaA* was deleted by P1 transduction of the *igaA*::kan allele in the presence of L-arabinose. Under permissive conditions, this strain was viable and Rcs activity was comparable to that measured in wild-type cells carrying the chromosomal copy of *igaA* (**S4 Fig**). After growing overnight under permissive conditions, cells were subjected to an initial depletion step by growing for ~8 generations in the presence of D-fucose, a non-metabolizable analog of L-arabinose which can be used to lower the expression levels from P_BAD_ [25]. In this case, IgaA became undetectable (**S5 Fig**; IgaA was detected by taking advantage of a penta His-tag present at the C-terminus). However, no growth defect was observed, consistent with the fact that IgaA efficiently represses Rcs even when expressed at low levels (**Fig 3A**). Only after the cells were sub-cultured in non-permissive conditions (in presence of D-fucose), the growth of the *igaA* depletion strain became severely affected (**Fig 3A**). As expected, decreased growth correlated with Rcs induction (**Fig 3B**)**.** In parallel experiments, cells in which IgaA had been initially depleted were serially diluted and spotted on LB-agar plates. Corroborating the results above, these cells could not grow on plates supplemented with L-fucose (non-permissive conditions) when they had a functional Rcs system, whereas Δ*rcsB* cells grew normally, thus confirming the essential role played by IgaA in inhibiting the Rcs system **(Fig 3C)**.

### The N-terminal, cytoplasmic domain of IgaA plays a key role in Rcs repression

**Fig 4:**
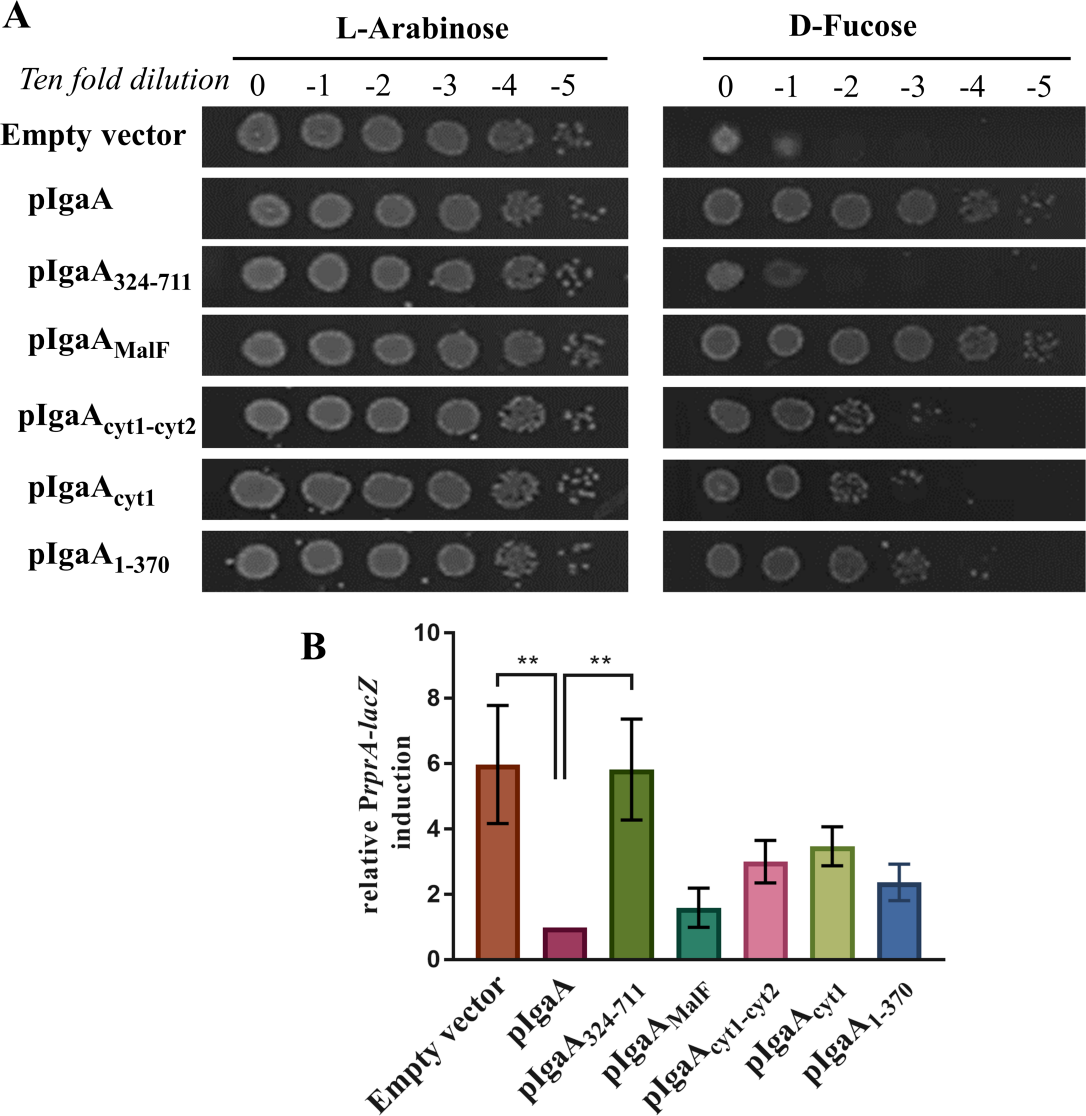
Key role of the IgaA N-terminal domain in Rcs system repression. (A) The IgaA N-terminal portion can rescue *E. coli* survival under non-permissive conditions. After initial igaA depletion, strains carrying empty pSC232 or expressing flag-tagged IgaA (from pSC238), IgaA_324-711_ (from pNH441), IgaA_MalF_ (from pNH692), IgaA_cyt1-cyt2_ (from pNH714), IgaA_cyt1_ (from pNH636) or IgaA_1-370_ (from pNH561) were serially diluted (10-fold dilutions) and spotted on LB-agar plates containing 100 μM IPTG and either 0.2% L-arabinose (permissive) or 0.2% D-fucose (non-permissive). Plates shown are representative of at least 3 independent experiments. (B) The IgaA N-terminal portion can partially repress the Rcs system under non-permissive conditions. Cells were inoculated after igaA initial depletion in LB medium-supplemented with 100 μM IPTG and 0.2% D-fucose. Rcs system activation was monitored after 6 hours. Values represent the mean of three biological replicates normalized to the corresponding strain treated with 0.2% L-arabinose. Error bars represent SEM.

To gain insights into the mechanism of action of IgaA, we generated a series of IgaA variants corresponding to different topological regions of this protein. We tested their ability, when expressed from an IPTG-dependent promoter carried on a plasmid, to control Rcs and complement the growth defect of the depletion strain under non-permissive conditions. IgaA_324-711_-fl, corresponding to the C-terminal periplasmic domain anchored to the IM *via* TMIV and TMV as explained above (**Fig 1C**), was tested first. However, as shown in **Fig 4**, it failed to rescue cell survival and had almost no repressing effect on Rcs. We next tested IgaA_MalF_-fl, an IgaA variant in which the C-terminal periplasmic domain is replaced by the periplasmic domain of MalF, the maltose transport system permease (**Fig 1C**). Thus, this variant (here referred as IgaA_MalF_-fl) lacks the periplasmic domain while keeping the cytoplasmic and membrane parts of IgaA intact. Remarkably, IgaA_MalF_-fl was able to fully rescue the growth of the depletion strain under non-permissive conditions (**Fig 4A**) and repressed Rcs activation to a level similar to that observed when wild-type IgaA was expressed (**Fig 4B**). Consistent with this, cells expressing IgaA_MalF_-fl remained viable after *igaA* deletion, only exhibiting a mild growth defect (**Table 1**). Thus, altogether, these data indicated that the inhibitory activity exerted by IgaA on the Rcs system does not depend on the periplasmic domain but rather on the cytoplasmic and membranous regions.

**Table 1:** Complementation of *igaA*::*kan* cells by IgaA variants.

Plasmid/IgaA variant	Growth on LB-agar
empty vector	-
IgaA*	+++
IgaA_MalF_*	++
IgaA_cyt1-cyt2_*	-
IgaA_cyt1_*	-
IgaA_1-370_*	-
IgaA_1-370_*+ pIgaA_324-711_**	+++

* IgaA variants expressed with C-terminal triple flag tag from the IPTG- inducible plasmids pSC238, pNH441, pNH692, pNH714, pNH636 and pNH561 in wild-type cells. The *igaA*::*kan* allele was then introduced using P1 transduction.

** IgaA variant expressed with a C-terminal penta-His tag from the arabinose-inducible pNH539.

+++ indicates normal growth, ++ mild growth impairment and − no growth.

To further zoom in on the portion of IgaA responsible for the inhibitory activity of this protein and directly investigate the importance of the cytoplasmic region, we then tested the impact of expressing the two cytosolic domains (IgaA_cyt1-cyt2_-fl, in which the two domains are joined by a disordered linker) on the growth of the depletion strain under non-permissive conditions. Expression of IgaA_cyt1-cyt2_-fl could partially rescue *igaA* lethality (**Fig 4A**) and repress Rcs (**Fig 4B**). Remarkably, similar results were obtained when the first N- terminal cytosolic domain (IgaA_cyt1_-fl) was expressed alone. Thus, the cytoplasmic portion of IgaA in general and the first cytosolic domain in particular appear to contribute to a large extent to the inhibitory activity of this protein.

### Co-expressing the N- and C-terminal domains of IgaA restores full inhibitory activity

Although significant, the impact of expressing IgaA_cyt1-cyt2_-fl or IgaA_cyt1_-fl on Rcs repression was, however, only partial (**Fig 4B**). Consistent with this, expression of these two IgaA variants did not allow *igaA* to be deleted from the chromosome (**Table 1)**. This led us to investigate the importance of anchoring the cytoplasmic domains of IgaA to the membrane. To that purpose, we generated IgaA_1-370_-fl_,_ a variant corresponding to the N-terminal portion of the protein comprised between TMI and TMIV. In this variant, both cytoplasmic domains are anchored to the IM. However, expression of IgaA_1-370_-fl did not substantially improve survival of the depletion strain compared to IgaA_cyt1-cyt2_-fl or IgaA_cyt1_-fl (**Fig 4A**). It also did not have a significant impact on Rcs repression compared to the cytosolic domains alone (**Fig 4B**) and did not allow the *igaA*::*kan* allele to be transduced **(Table 1)**. Thus, anchoring the cytoplasmic domains to the membrane does not significantly increase their ability to repress Rcs.

**Fig 5:**
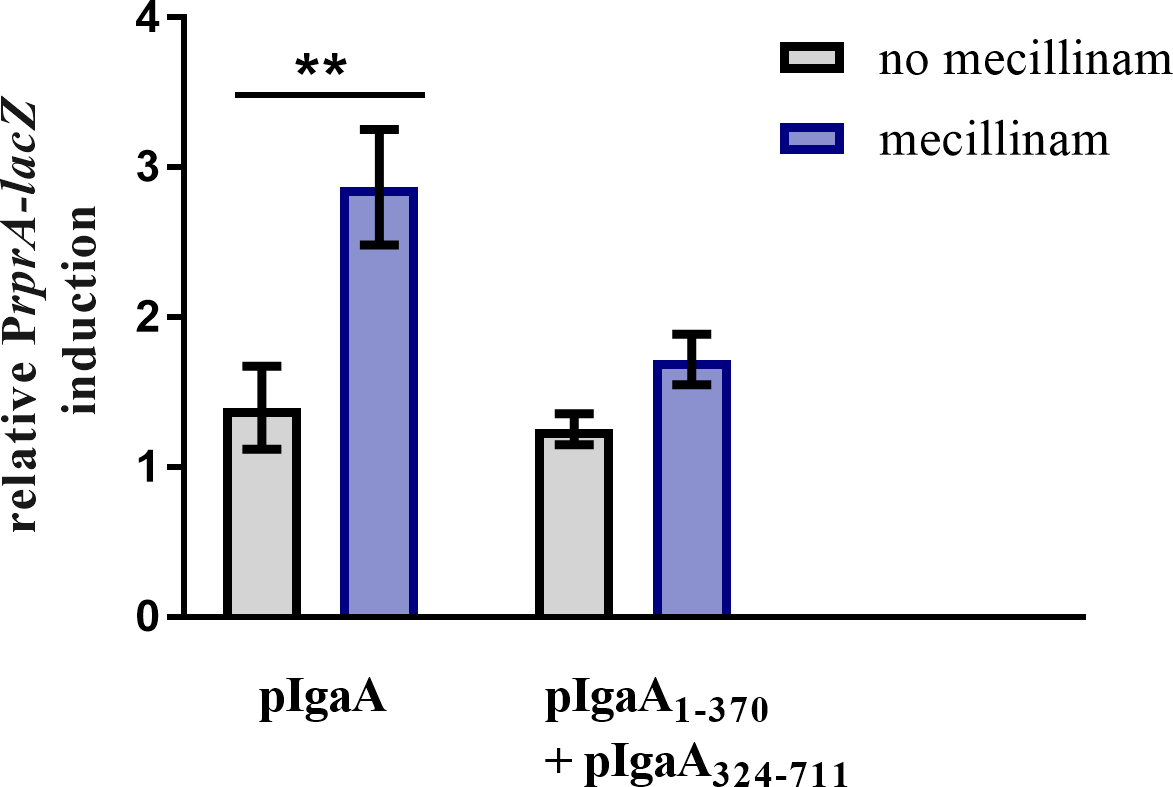
Reconstitution of IgaA fully restores Rcs system repression. *igaA*::*kan* cells co-expressing IgaA_1-370_-fl (from pNH565) and IgaA_324-711_-His (from pNH539) or expressing IgaA-fl (form pSC238) were grown in presence of 0.2% L-arabinose and 100 μM IPTG until mid-log phase. Rcs system activation was then monitored as described in **Fig 2B**. Values represent the mean of three biological replicates normalized to wild-type DH300 cells transformed with the empty vectors (pNH431 and pSC232) and treated similarly. Error bars represent SEM.

This led us to investigate whether full IgaA inhibitory activity could be recovered by co-expressing its N- and C-terminal portions. Excitingly, we found that co-expression of IgaA_1-370_-fl and IgaA_324-711_-His allowed deletion of the chromosomal copy of *igaA* (**Table 1**) and fully repressed Rcs (**Fig 5**). Thus, although the N-terminal domain of IgaA is crucially important for tuning down Rcs, complete inhibition can only be achieved when the C-terminal domain is co-expressed (see Discussion). This reconstituted IgaA could not, however, respond to cues that induce Rcs in an RcsF-dependent manner (**Fig 5**).

## Discussion

Because of the crucial importance of the envelope for growth and survival, bacteria invest a great deal in sensing perturbations that occur in that compartment. In *E*. *coli*, several stress-sensing systems cooperate in monitoring envelope integrity. Investigating how bacterial cells sense and respond to envelope stress will reveal how they efficiently integrate different types of stress signals and rapidly convey the information from the envelope to the cytoplasm, where cellular behavior is controlled. It will also contribute to elucidating how bacteria coordinate signal sensing with envelope growth and assembly.

Rcs, which senses defects in OM and PG integrity, is a particularly intricate envelope surveillance system. It is probably because of this complexity that, despite decades of research on Rcs, our understanding of its functioning remains incomplete. For instance, although major insights into how the lipoprotein RcsF detects stress in the outer part of the envelope were recently reported [11,12,26], we still only partially understand the sensing mechanism. In addition, a couple of genetic perturbations have been shown to activate Rcs independently of RcsF, but the mechanism of action is completely unknown [5,27].

Here, we investigated the IM protein IgaA in order to understand how this auxiliary Rcs component receives stress signals and controls the phosphorelay. Other proteins that regulate two-component systems have been described, such as *E*. *coli* CpxP, a periplasmic protein that controls the activity of the Cpx system [28,29]. However, in comparison to most two-component system fine-tuners, IgaA is particularly intriguing for at least two reasons. First, IgaA is essential [20]. Second, whereas two-component systems regulators are usually small proteins located either in the periplasm or in the cytoplasm [29,30], IgaA is a polytopic membrane protein with large cytoplasmic and periplasmic domains [16,31], suggesting that it regulates signal sensing and transduction by integrating molecular information in both cellular compartments. Thus, the unusual features of IgaA point to a unique and particularly complex mode of action.

We and other previously reported the ability of RcsF to interact with the periplasmic domain of IgaA when this latter was expressed as a soluble protein [11,22]. However, the physiological importance of this interaction remained unclear. Here, we clearly demonstrated that RcsF interacts with the full-length, IM-anchored IgaA and that the interaction increases in response to stress. These data both establish the functional relevance of the IgaA-RcsF interaction and provide strong experimental support to the model that RcsF induces Rcs by interacting with IgaA. They also confirm the role of the periplasmic domain of IgaA in being the primary receiver of the signal sensed and transmitted by RcsF. Finally, they are consistent with our recent finding that preventing the RcsF-IgaA interaction by increasing the periplasmic size does not allow Rcs system activation in response to stress [32]. Interestingly, results from ribosomal profiling experiments [23] indicated that RcsF (~3,100 copies/cell) is in large excess over IgaA (~200 copies/cell), thus predicting that if less than 10% of the pool of RcsF molecules interact with IgaA, full Rcs activation will be observed. Remarkably, this is exactly what we previously showed by using a version of RcsF that remains soluble in the periplasm and is therefore not sequestered away from IgaA by its β-barrel partners in the OM: Rcs was fully activated when this soluble version of RcsF was expressed at ~10% of the of the wild-type RcsF levels [11]. This further highlight and confirms the pivotal role played by the IgaA-RcsF interaction in controlling Rcs activity.

We also observed that the IgaA-RcsF interaction occurs even in non-stressed cells, in which basal Rcs activity is measured. Under normal conditions, RcsF is occluded from IgaA by its OM partners [11]. We therefore think that the IgaA-RcsF complex that is detected in the absence of stress (and that is responsible for basal Rcs activity) involves the small fraction of the pool of RcsF molecules that is found in the IM when *E*. *coli* membranes are fractionated on sucrose density gradients [11]. This fraction is likely constituted by newly synthesized RcsF molecules waiting to be extracted from the IM by the LolCDE complex and transferred to the chaperone LolA for transport to the OM [11]. Thus, if the activity of the Lol system is impaired following envelope stress, RcsF will likely accumulate in the IM, turning on Rcs. Interestingly, Rcs induction leads to higher *lolA* expression [33], which might help to overcome the damage. It is therefore possible that one of the roles of the Rcs system is to monitor lipoprotein trafficking across the cell envelope, as previously suggested [11].

While stress is first sensed by RcsF in the outer part of the envelope, the reactions of the phosphorelay occur in the cytoplasm. With domains located in both cellular compartments, IgaA appears therefore to be well equipped to play a direct role in transducing stress signals across the IM. Supporting this idea, we showed that while the C-terminal periplasmic domain of IgaA serves as the primary signal receiver, the N-terminal cytoplasmic domains, and the first cytoplasmic domain in particular, play an important role in inhibiting Rcs. It is therefore possible that formation of the RcsF-IgaA interaction in the periplasm triggers conformational rearrangements in the cytosolic part of IgaA that, as a result, impact the inhibitory function of this protein on the phosphorelay **(S6 Fig)**. The fact that co-expressing the N-terminal and C-terminal parts of IgaA, while restoring full Rcs inhibition, fails to reconstitute a protein able to trigger Rcs under stress supports the idea that IgaA is involved in signal transduction across the IM and indicates that the transfer of information across the membrane requires a full-length, intact polypeptide. Interestingly, it was recently found that the redox state of cysteine residues located in the periplasmic domain of *S*. *enterica* IgaA was altered by a mutation in the cytosolic domain of this protein, thus further highlighting the functional connection between the two parts of IgaA [31].

Although nothing is known on how IgaA_cyt1_ down-regulates Rcs, it is most likely by interacting with one or more of the downstream components of the phosphorelay. In the absence of stress, IgaA_cyt1_ could, for instance, interact with RcsC to alter the phosphatase/kinase balance or perturb the phosphotransfer reaction between RcsC and RcsD or RcsD and RcsB. Interestingly, IgaA_cyt1_ exhibits significant structural similarity to the OB fold (oligonucleotide/oligosaccharide binding motif), a fold often found in domains involved in protein-protein interaction and nucleotide binding [34]. Future work is required to understand in detail how IgaA_cyt1_ inhibits Rcs.

Our results also show that, although important, the cytoplasmic part of IgaA is not sufficient for full Rcs repression, which, indeed, requires co-expression of the C-terminal portion. It is interesting that a more potent repression was observed in cells expressing IgaA_MalF_ but not in those expressing IgaA_1-370_ (**Table 1** and **Fig 4B**). Indeed, the only segment of the IgaA sequence that is present in IgaA_MalF_ but absent in IgaA_1-370_ is the C-terminally located TMV. Thus, this result suggests that TMV may also play a role in down-regulating Rcs.

Future work is also required to investigate how RcsC and RcsD are interconnected to the other Rcs components. Indeed, although the role of these two IM proteins in the phosphorelay is well established, nothing is known on how they are regulated. We also do not know to what extent they participate in signal sensing. As discussed above, our results suggest that IgaA_cyt1_ could interact with the cytoplasmic domain of RcsC and/or RcsD. In addition, both RcsC and RcsD display large periplasmic domains whose function remains elusive.

A recent report suggests that the periplasmic domain of RcsC interacts with RcsF, but the functional role of this potential interaction remains to be shown [22]. It is possible that, by interacting with the periplasmic domain of RcsC (and perhaps also RcsD), RcsF influences how these proteins are inhibited by IgaA. Alternatively, an RcsF-RcsC interaction might also contribute to fine-tuning the activity of the Rcs system. It is also possible that additional proteins further modulate Rcs signaling, such as YfgM, a single pass IM protein, that was suggested to work as an anti-RcsB factor, but whose mechanism of action remains unknown [35]. Dissecting the interplay between different Rcs components and understanding how they cooperate in integrating stress signals will likely prove to be a complex and challenging task.

## Materials and methods

### Media, bacterial strains and plasmids

The bacterial strains used in this study are all derivatives of *E*. *coli* MG1655 carrying a chromosomal *rprA*::*lacZ* fusion at the lambda attachment site (DH300) [36]. All derivatives used are listed in **S1 Table**. Bacterial cells were cultured using LB-Miller at 37°C containing (whenever necessary) the following concentration of antibiotics: chloramphenicol (25 μg/μl), spectinomycin (100 μg/ml) and kanamycin (50 μg/μl). When two antibiotics were combined, half of the mentioned concentrations were used.

Except for *igaA*::*kan* mutations, all null alleles were generated from the corresponding single deletion mutants in the Keio collection [37] and transferred to the wild-type DH300 strain using P1 phage transduction. All generated mutants were checked by PCR. For excision of the kanamycin resistance cassette, we used the pCP20 plasmid [38].

Plasmids used in this study are all derived from pNH401 (pBAD33-based) or pSC232 (pAM238-based) and are listed in **S2 Table**. For cloning purposes, standard molecular biology techniques were followed, using KOD polymerase (Novagen), restriction enzymes (New England Biolabs) and XL-1 blue as cloning strain. Chromosomal DNA from MG1655 was used as a template DNA. The sequences of the primers used for cloning and checking gene replacement are available upon request.

### β-galactosidase assays

β-galactosidase assays were performed according to the modified Miller assay as described previously [32,39].

### Construction and phenotypic assessment of the *igaA* depletion strain

We first generated an *igaA*::kan mutant in the *rcsB* mutant of the Keio collection (in which *igaA* is dispensable; the kanamycin cassette had previously been excised). To that purpose, the Δ*rscB* mutant, harboring pKD46, was transformed with a PCR product corresponding to the kanamycin cassette flanked by 50 bp *igaA* up- and downstream its genomic locus [38], generating, after recombination, strain SEN549. The *igaA*::*kan* allele from strain SEN549 was then P1 transduced into DH300 cells harboring pNH586 (pBAD33 with IgaA-His) in the presence of 0.2% L-arabinose. This strain was renamed NH594. In order to deplete IgaA-His_,_ an initial *igaA* depletion was performed by growing NH594 overnight in presence of the corresponding antibiotic and 0.2% L-arabinose. The cells were then washed three times with arabinose- free medium and diluted 1/1000 in LB-Miller broth containing 0.2% D-fucose until an OD_600_ of 0.8–1, yielding *igaA*-depleted cells. The cells were then washed thoroughly with LB, serially diluted and spotted on LB-Miller-agar plates supplemented either with 0.2% L-arabinose or 0.2% D-fucose. Alternatively, the *igaA*-depleted cells were inoculated in fresh LB-Miller supplemented with either 0.2% L-arabinose or 0.2% D-fucose and the growth was monitored by measuring the optical density (OD) at 600 nm every hour. At the indicated intervals, aliquots were saved to monitor Rcs system activation by β-galactosidase assay and protein expression levels by western blotting.

The same protocols were followed to assess the ability of the different IgaA variants to complement *igaA* depletion in strains expressing pSC232, pSC238, pNH441, pNH561, pNH692, pNH714 or pNH636 in NH594. In this case, 0.2% glucose was added to repress both arabinose and IPTG-inducible promoters in the initial depletion, while 100 μM IPTG was added to induce expression of the abovementioned plasmids.

### Growth curves

Growth curves (without prior *igaA* depletion) were constructed by growing the corresponding strains overnight in presence of 0.2% L-arabinose and 100 μM IPTG (if required), then diluting 1/1000 in fresh media. The growth was monitored by measuring OD at 600 nm.

### *In vivo* DTSSP crosslinking and flag tag immunoprecipitation

*In vivo* DTSSP crosslinking was performed as previously described [11] with some modifications. Briefly, strains expressing pSC238 or pNH441 were grown in presence of 100 μM IPTG until late log phase. Whenever needed, mecillinam at a final concentration of 0.3 μg/ml was added when the cells reached an OD_600_ of 0.2 and incubated for one hour. The cells were then washed with PBS, pH 7 and treated with 200 μM of DTSSP (Covachem) for one hour at 30°C. Following quenching with 100 mM glycine, the cells were TCA precipitated and dissolved in 5X non-reducing Laemmli buffer at 60°C before dilution with TBS buffer containing 0.2% n-Dodecyl-β-D-Maltoside (DDM) and incubated overnight with Flag-conjugated beads (Sigma). After three washing steps, the proteins were eluted with 100 mM glycine, pH 2, containing 0.2% DDM and then subjected to western blot analysis.

### Western blotting

Aliquots from growing cultures were TCA precipitated and solubilized by heating at 60°C with 1X non-reducing Laemmli buffer. Eluted samples after immunoprecipitation were prepared similarly after measuring protein concentration but without TCA precipitation. The samples were loaded on precast NuPAGE Bis-Tris gels (Thermo). Transfer was performed using standard semi-dry transfer method on nitrocellulose membrane (Thermo) and the membranes were blocked using 5% non-fat dry milk. Primary antibodies were used at the following dilutions: anti-RcsF (1:2,000), anti-flag (Sigma 1:3,000), anti-PtsI (1:30,000) anti-His (Qiagen, 1:8,000). Horse-radish peroxidase-conjugated secondary antibody was used at a concentration of 1:10,000 or 1:20,000 and the membranes were developed using ECL (Thermo) or ECL-Prime (GE healthcare), respectively. Chemiluminescence signal was detected on Fuji X-ray films.

### Figure preparation, data normalization and statistical analysis

Curves and bar charts represent an average of at least three biological replicates and were prepared using Prism 6 (Graph-Pad Software, Inc.). Statistical analysis was performed using the same software. Statistical significance was calculated based on two- way ANOVA tests for all experiments except for Fig 4B where one-way ANOVA was used.

## Supporting information

S1 Fig Expression of IgaA-fl complements an *igaA*::Kan strain. Expression of IgaA-fl (from pSC238) was induced with 100 μM IPTG and growth was monitored at OD_600_ in LB-Miller media. The growth was similar to the wild-type DH300 strain carrying the empty plasmid (pSC232).
(TIF)

S2 Fig IgaA-fl and IgaA_324-711_-fl localize to the membrane fraction of *E*. *coli*. S2A: Expression of IgaA-fl (from pSC238) was induced with 100 μM IPTG until late log phase. The membrane fractions were separated using two successive ultracentrifugation steps and solubilized in 2% DDM. S2B: Expression of IgaA-fl_324-711_ (from pNH441) was induced with 100 μM IPTG until late log phase. The membrane fractions were treated as described in S2A. For both panels: Black arrows indicate the specific bands detected by the antibody used. W: whole cell lysate (lane 1), S: soluble fraction (lanes 2), M: solubilized membrane fraction (lane 3) and I: insoluble fraction (lane 4). DsbD and DsbA were used as controls for the membrane and soluble fractions, respectively.
(TIF)

S3 Fig Basal Rcs activity as detected in wild-type cells in the absence of stress. *PrprA- lacZ* activity is two-fold higher in the wild type than in *ΔrcsB* cells. Wild-type and *ΔrcsB* cells carrying the empty vector (pSC232) or expressing IgaA-fl (from pSC238) were grown in the presence of 100 μM IPTG until mid-log phase and Rcs activity was monitored as previously [36].
(TIF)

S4 Fig Viability and Rcs system repression of the *igaA* depletion strain under permissive conditions. When grown in 0.2% L-arabinose (permissive conditions), *igaA*::*kan* cells expressing IgaA-His (from pNH586) show comparable growth (A) and Rcs system activity (B) to wild-type DH300 cells harboring the empty vector.
(TIF)

S5 Fig IgaA-His levels are undetectable in the *igaA* depletion strain after growth under non-permissive conditions. After growing for ~8 generations in presence of 0.2% L-arabinose (permissive conditions) or 0.2% D-fucose (non- permissive conditions), IgaA-His is similarly expressed or depleted in both *igaA*::*kan* (lanes 1 and 3) and *ΔrcsB igaA*::*kan* strains (lanes 2 and 4). Cells were precipitated with trichloroacetic acid, normalized according to their respective OD_600_ and loaded for western blot analysis. Antibody raised against PtsI (a cytoplasmic protein unrelated to the Rcs system) was used as a loading control.
(TIF)

S6 Fig IgaA receives stress signals via its C-terminal periplasmic domain and inhibits downstream Rcs components mostly via its first cytosolic domain. Left panel: Under normal (non-stress) conditions, IgaA, predominantly through its first N-terminal cytosolic domain, represses the Rcs system, likely by interacting with either RcsC or RcsD or both (dotted square), *via* an unknown mechanism. Right panel: When cells are exposed to envelope damage (Rcs inducing cues), newly synthesized RcsF molecules interact with the C-terminal periplasmic domain of IgaA. This interaction relieves the inhibition that IgaA exerts on the downstream Rcs components, leading to Rcs activation. In both panels, OM-anchored RcsF in complex with its β-barrel partners is not represented.
(TIF)

S1 Table Strains used in this study. (DOCX)

S2 Table Plasmids used and generated in this study. (DOCX)
