# Supplementary figures and images for: Distinct domains of Escherichia coli IgaA connect envelope stress sensing and down-regulation of the Rcs phosphorelay across subcellular compartments
Source: PLoS Genet. 2018 May 31;14(5):e1007398. doi: 10.1371/journal.pgen.1007398 (PMC5978795; doi:10.1371/journal.pgen.1007398)

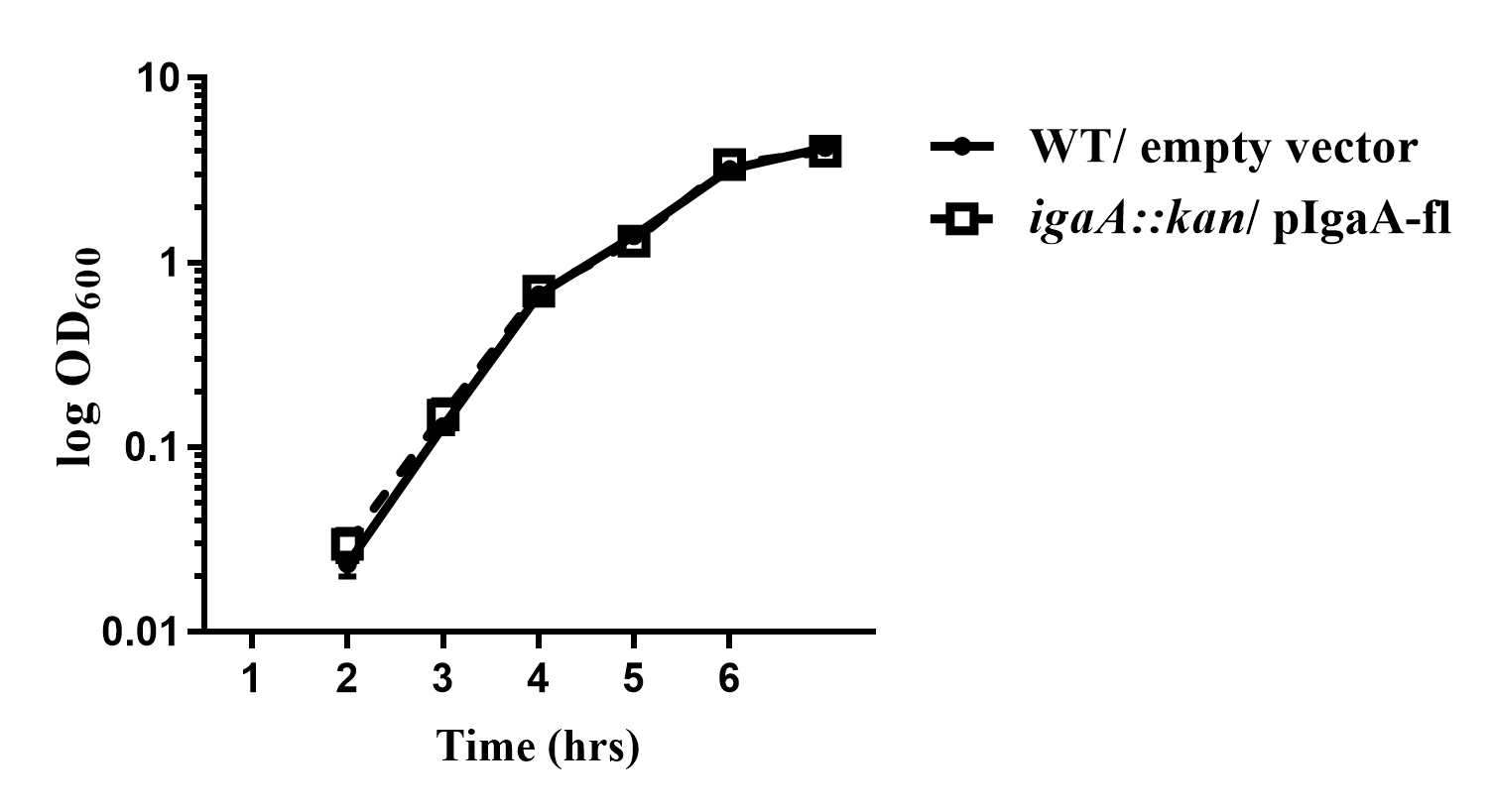

Supplement: S1 Fig — Expression of IgaA-fl (from pSC238) was induced with 100 μM IPTG and growth was monitored at OD600 in LB-Miller media. The growth was similar to the wild-type DH300 strain carrying the empty plasmid (pSC232). (TIF) [file pgen.1007398.s001.tif]

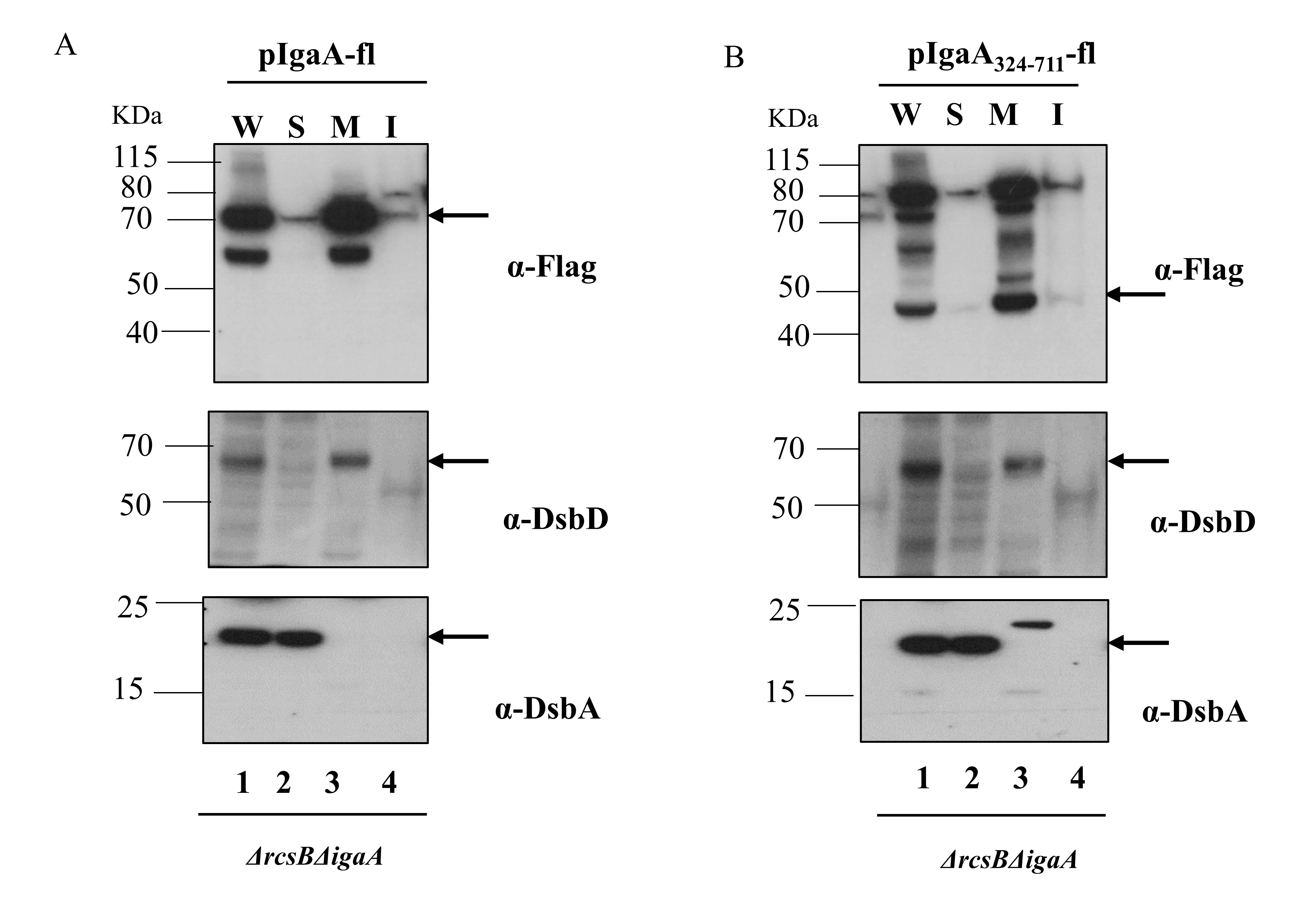

Supplement: S2 Fig — S2A: Expression of IgaA-fl (from pSC238) was induced with 100 μM IPTG until late log phase. The membrane fractions were separated using two successive ultracentrifugation steps and solubilized in 2% DDM. S2B: Expression of IgaA-fl324-711 (from pNH441) was induced with 100 μM IPTG until late log phase. The membrane fractions were treated as described in S2A. For both panels: Black arrows indicate the specific bands detected by the antibody used. W: whole cell lysate (lane 1), S: soluble fraction (lanes 2), M: solubilized membrane fraction (lane 3) and I: insoluble fraction (lane 4). DsbD and DsbA were used as controls for the membrane and soluble fractions, respectively. (TIF) [file pgen.1007398.s002.tif]

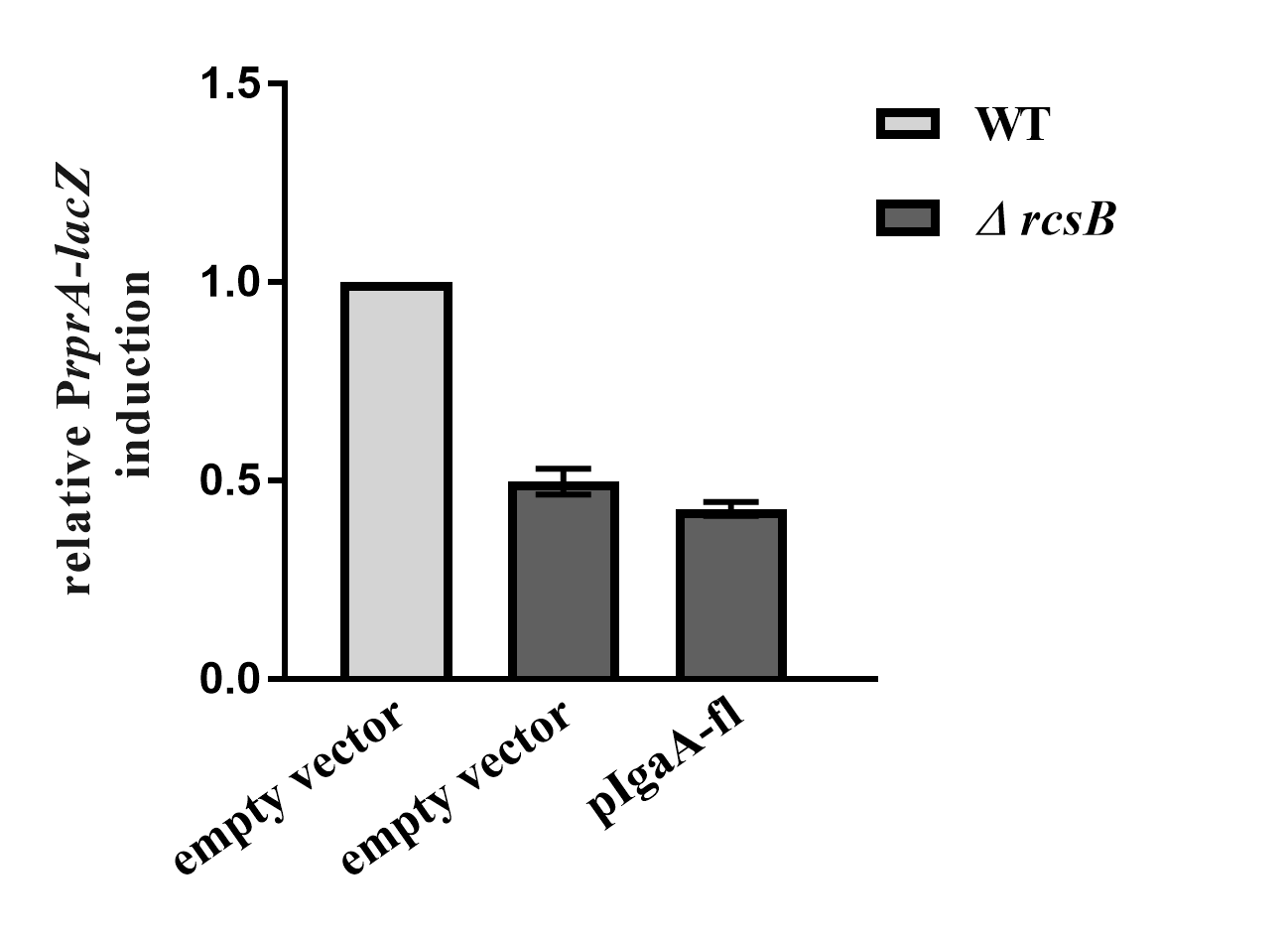

Supplement: S3 Fig — PrprA- lacZ activity is two-fold higher in the wild type than in ΔrcsB cells. Wild-type and ΔrcsB cells carrying the empty vector (pSC232) or expressing IgaA-fl (from pSC238) were grown in the presence of 100 μM IPTG until mid-log phase and Rcs activity was monitored as previously [36]. (TIF) [file pgen.1007398.s003.tif]

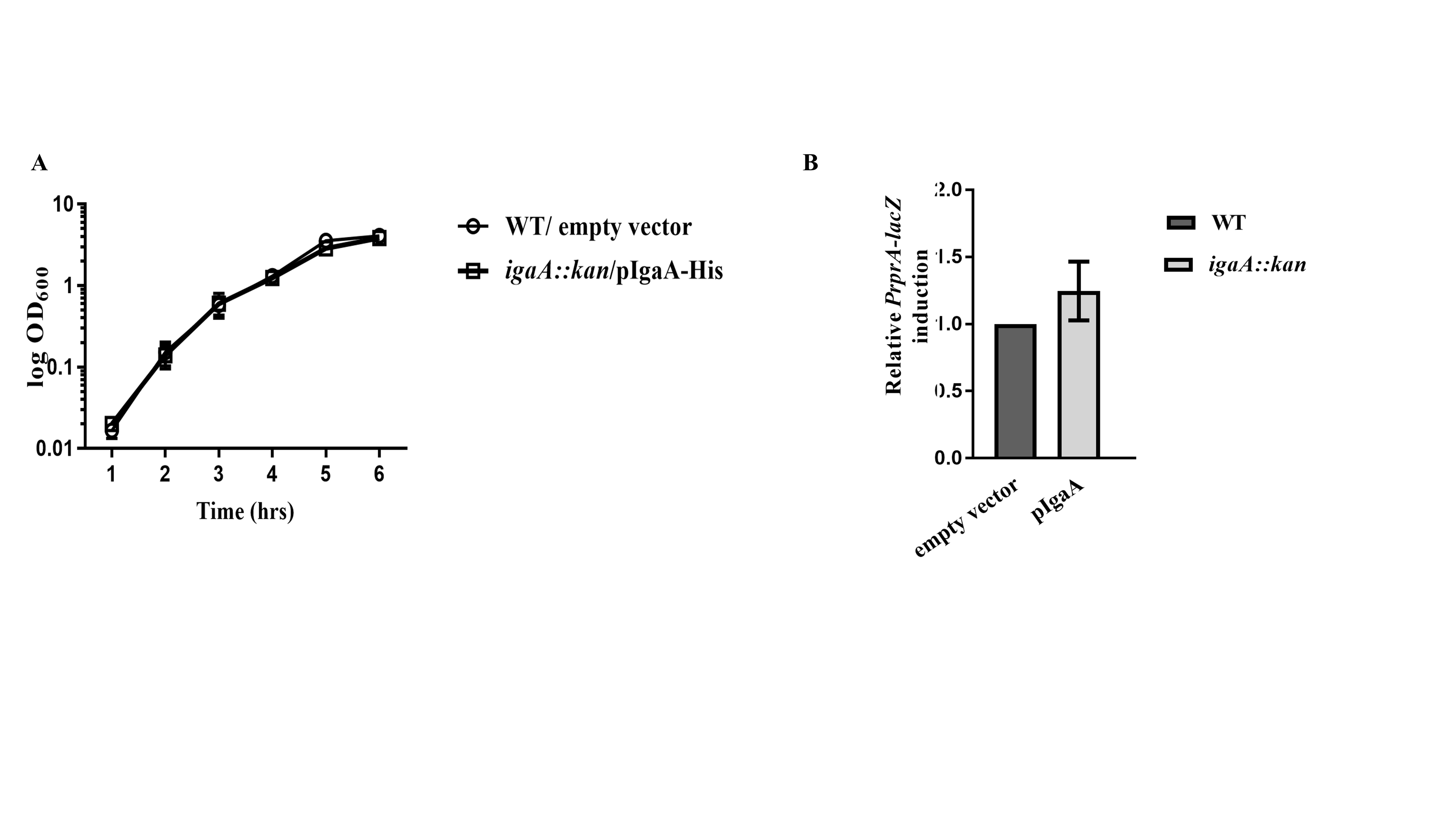

Supplement: S4 Fig — When grown in 0.2% L-arabinose (permissive conditions), igaA::kan cells expressing IgaA-His (from pNH586) show comparable growth (A) and Rcs system activity (B) to wild-type DH300 cells harboring the empty vector. (TIF) [file pgen.1007398.s004.tif]

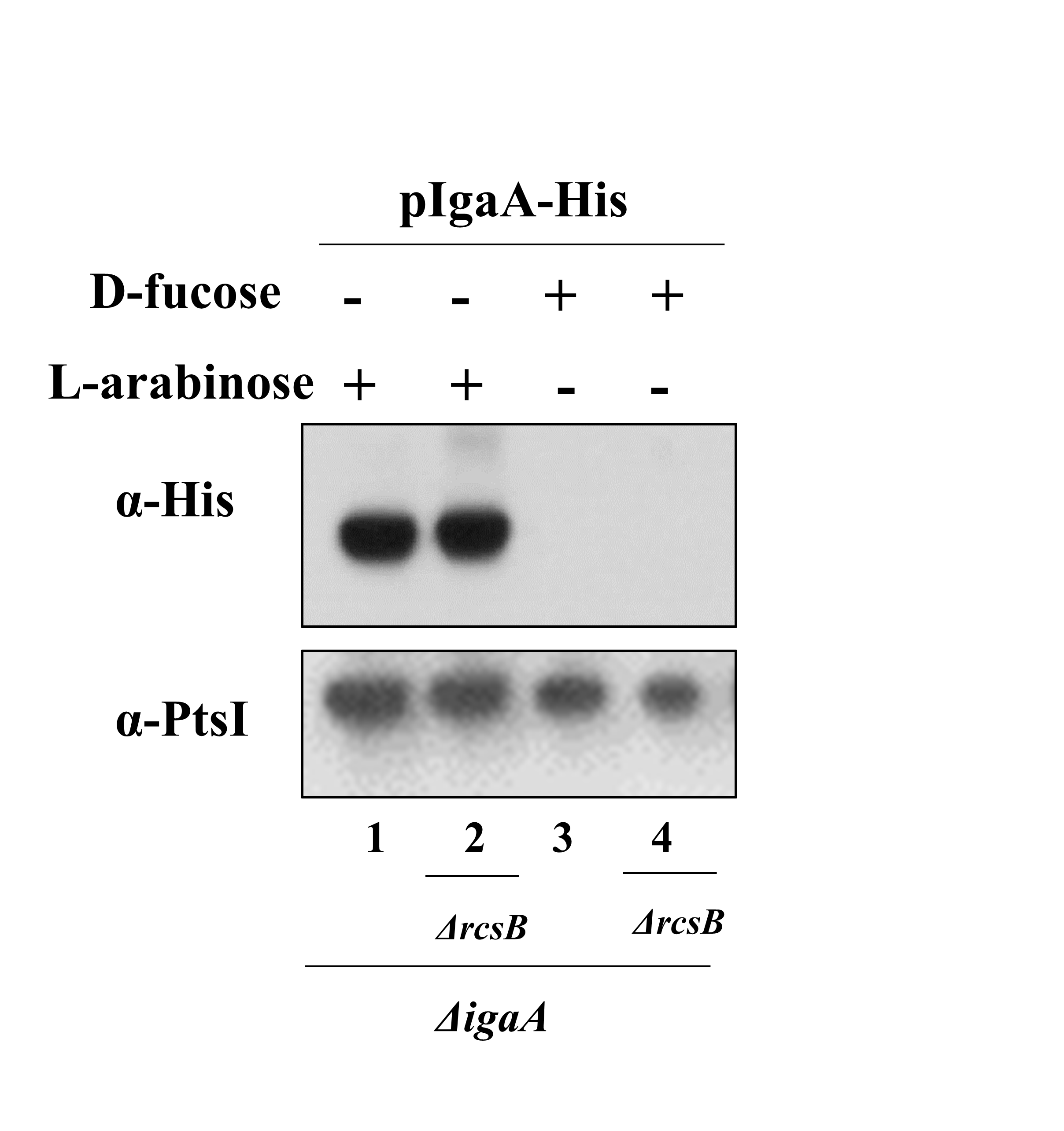

Supplement: S5 Fig — After growing for ~8 generations in presence of 0.2% L-arabinose (permissive conditions) or 0.2% D-fucose (non- permissive conditions), IgaA-His is similarly expressed or depleted in both igaA::kan (lanes 1 and 3) and ΔrcsB igaA::kan strains (lanes 2 and 4). Cells were precipitated with trichloroacetic acid, normalized according to their respective OD600 and loaded for western blot analysis. Antibody raised against PtsI (a cytoplasmic protein unrelated to the Rcs system) was used as a loading control. (TIF) [file pgen.1007398.s005.tif]

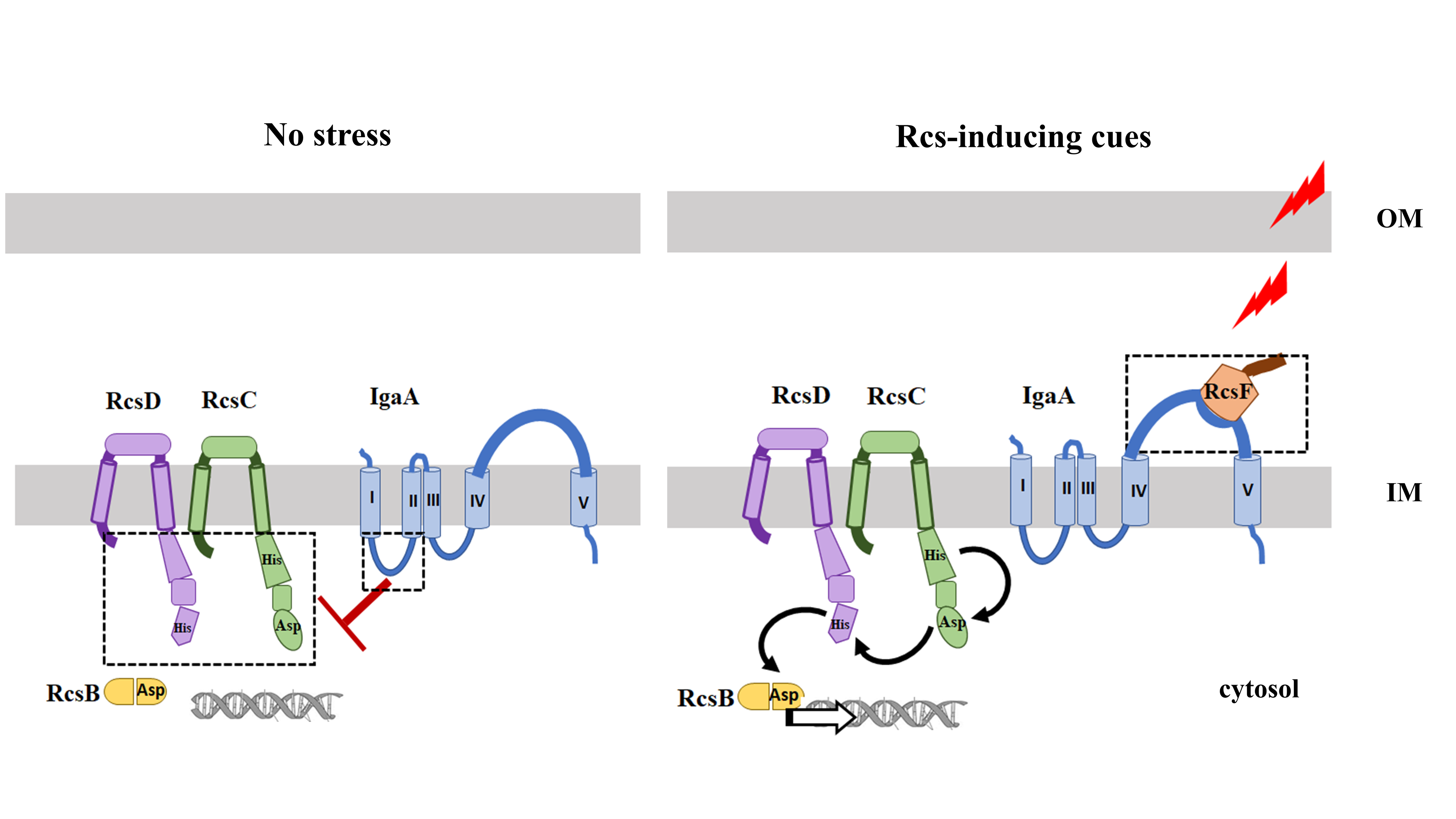

Supplement: S6 Fig — Left panel: Under normal (non-stress) conditions, IgaA, predominantly through its first N-terminal cytosolic domain, represses the Rcs system, likely by interacting with either RcsC or RcsD or both (dotted square), via an unknown mechanism. Right panel: When cells are exposed to envelope damage (Rcs inducing cues), newly synthesized RcsF molecules interact with the C-terminal periplasmic domain of IgaA. This interaction relieves the inhibition that IgaA exerts on the downstream Rcs components, leading to Rcs activation. In both panels, OM-anchored RcsF in complex with its β-barrel partners is not represented. (TIF) [file pgen.1007398.s006.tif]
