# Supplementary material for: Distinct domains of Escherichia coli IgaA connect envelope stress sensing and down-regulation of the Rcs phosphorelay across subcellular compartments
Source: PLoS Genet. 2018 May 31;14(5):e1007398. doi: 10.1371/journal.pgen.1007398 (PMC5978795; doi:10.1371/journal.pgen.1007398)
Supplement: S1 Table — (DOCX) [file pgen.1007398.s007.docx]

| **Strain** | **Genotype or features** | **Source or reference** |
| --- | --- | --- |
|  |  |  |
| DH300 | *rprA-lacZ* MG1655 (argF-lac)U169 | [36] |
| NH246 | DH300 *igaA::kan* pSC238 | This study |
| NH594 | DH300 *igaA::kan* pNH586 | This study |
| NH575 | DH300 *igaA::kan* pNH539+ pNH561 | This study |
| NH365 | DH300 Δ *rcsBΔ igaA* | This study |
| NH694 | DH300 Δ *rcsFΔ rcsBΔ igaA* | This study |
| SEN549 | Keio *Δ rcsB* *igaA::kan* | This study |
