# Supplementary material for: Distinct domains of Escherichia coli IgaA connect envelope stress sensing and down-regulation of the Rcs phosphorelay across subcellular compartments
Source: PLoS Genet. 2018 May 31;14(5):e1007398. doi: 10.1371/journal.pgen.1007398 (PMC5978795; doi:10.1371/journal.pgen.1007398)
Supplement: S2 Table — (DOCX) [file pgen.1007398.s008.docx]

| **Plasmids** | **Features** | **Source or reference** |
| --- | --- | --- |
|  |  |  |
| pAM238 | IPTG-regulated Plac, pSC101-based, spectinomycin | (1) |
| pSC232 | modified pAM238^1^ | This study |
| pBAD33 | Arabinose- regulated, pACYC184-based, chloramphenicol | (2) |
| pNH431 | modified pBAD33^2^ | This study |
| pCP20 | FLP+ , λ cI857+ , λ PR Repts, ampicillin, chloramphenicol | (3) |
| pNH441* | pSC232 with IgaA (S324- E711)-3Xflag | This study |
| pSC238* | pSC232 with IgaA -3Xflag | This study |
| pNH561* | pSC232 with IgaA(M1-W370)-3Xflag | This study |
| pNH636* | pSC232 with IgaAcyt1 (K24-R204) -3Xflag | This study |
| pNH692* | pSC232 with IgaA(Δ361-655)-MalF(93-275) -3X flag | This study |
| pNH714* | pSC232 with IgaAcyt1-cyt2 (K23 to R204-G(SGGG)4SHM- A247 to R339)-3Xflag | This study |
| pNH586** | pNH431 with IgaA-5XHis | This study |
| pNH539** | pNH431 with IgaA (S324- E711)-5XHis | This study |

^1^ We modified pAM238 by inserting *lacIq* and the *trc* promoter from pTrc99a plasmid. We further modified the -10 region of the *trc* promoter and *lac* operator to weaken its basal activity, generating pSC232.

^2^ We modified pBAD33 by deleting its *Xba*I site.

* Genes were cloned in the plasmid pSC232 preceded by *igaA* native RBS between *Nco*I and *Xba*I restriction sites. The 3Xflag tag was inserted between *Xba*I and *Kpn*I sites at the C‑terminus. ** Genes were cloned in the plasmid pNH431 preceded by *igaA* native RBS between the plasmid *Sac*I site and an introduced *Xba*I site at the gene C-terminus. The 5XHis tag was introduced between the *Xba*I site and the plasmid *Kpn*I site.

**References:**

1. Dominique G, Bouché JP. ColE1-type vectors with fully repressible replication. Gene. 1991;105(1):17–22. https://doi.org/10.1016/0378-1119(91)90508-9 PMID:1937005

2. Guzman L-M, Belin D, Carson MJ, Beckwith J. Tight Regulation, Modulation, and High-Level Expression by Vectors Containing the Arabinose PBAD Promoter. J Bacteriol. 1995;177(14):4121–30. doi: 10.1128/jb.177.14.4121-4130.1995 PMID:7608087

3. Cherepanov, P, Wackernage, W. Gene disruption in Escherichia coli: TcR and KmR cassettes with the option of Flp-catalyzed excision of the antibiotic-resistance determinant. Gene. 1995;1995(158):9–14. https://doi.org/10.1016/0378-1119(95)00193-A PMID:7789817
